# Characterization of the complete chloroplast genome of *Scorpiothyrsus erythrotrichus* (Melastomataceae), an endemic to Hainan

**DOI:** 10.1080/23802359.2021.1997657

**Published:** 2021-11-18

**Authors:** Hong-Xin Wang, Lang-Xing Yuan, Qing-Hui Sun, Ai-Fang Weng, Yu-Wei Nian, Wen-Shu Chen, Qi-Xuan Meng

**Affiliations:** a Zhai Mingguo Academician Work Station, Sanya University, Sanya, China; b Institute Arts, Sanya University, Sanya, China; c School of Tropical Medicine and Laboratory Medicine, Hainan Medical University, Haikou, China

**Keywords:** Complete chloroplast, genome, phylogeny, Melastomataceae, *Scorpiothyrsus*

## Abstract

*Scorpiothyrsus erythrotrichus* belongs to Melastomataceae. Here, we present its complete plastome. To our knowledge, this is the first reported complete chloroplast genome of *S. erythrotrichus*. The complete plastome of *S. erythrotrichus* is 160,731 bp in length with a typical quadripartite structure, consisting of four regions: large single-copy (LSC) region (85,483 bp), small single-copy (SSC) region (17,007 bp), and two inverted repeat regions (IRs, 26,780 bp). It contains 128 genes (79 coding genes, four rRNAs, and 30 tRNAs). The overall GC content is 36.9% and in the LSC, SSC, and IR regions are 34.70%, 30.40%, and 42.50%, respectively. Our study contributes to the molecular phylogenetic studies of *Scorpiothyrsus* and Melastomataceae.

*Scorpiothyrsus erythrotrichus* (Merrill & Chun) H. L. Li, a shrublets with sparsely hispid, occurs in sparse to dense forests, mountain slopes, shaded places with an altitude of 600–1400 m, it is an endemic species to Hainan and included in threatened species list of China’s higher plants as critically endangered categories. Due to its spotted leaves, *S. erythrotrichus* has the potential for horticultural applications. With the rapid changes in climate and intensification of human activities, the distribution of *S. erythrotrichus* is rapidly reduced in recent decades. Understanding the spatial genetic pattern and demographic dynamics of the species can provide important guidelines for the protection and utilization of endemic species. *Scorpiothyrsus* is originally described as part of *Phyllagathis* (Merrill and Chun 1940) and later segregated as an independent genus based on the scorpioid paniculate inflorescences (Li 1944). However, Hansen (1992) noticed that *P. cymigera* shared some general resemblance with *Scorpiothyrsus*. Their close relationship is confirmed by phylogenetic data with rather strong support (Zhou et al. 2019). *Scorpiothyrsus* includes about three species and is mainly distributed in Guangxi and Hainan (Chen and Renner 2007). Although two complete plastid genomes of the genus have been reported, the plastome of *S. erythrotrichus* was not involved and genome features of the genus are still unclear because it is distributed scarce in the field. In this study, the complete plastid genome of *S. erythrotrichus* was sequenced to provide basic plastome features of *Scorpiothyrsus*, which will provide a valuable resource for further genetic conservation, evolution, and molecular breeding studies in the genus *Scorpiothyrsus*.

The sample of *S. erythrotrichus* was collected from Murui Mountain, Ding’an County, Hainan (110.288°N, 19.26°E, elevation 612 m). Fresh leaves were put into silica gel to preserve until DNA extraction. The voucher specimens were deposited in the herbarium of Sanya University (collector and collection number: Lang-xing Yuan (Langxingyuan@sanyau.edu.cn), BLHMJHD1). Genomic DNA of *S. erythrotrichus* was extracted according to CTAB method (Doyle and Doyle 1987). Total DNA was used to generate libraries with an average insert size of 350 bp, which were sequenced using the Illumina Hiseq-2500 platform at BGI-Shenzhen. Approximately, 14.0 GB of raw data were generated with 150 bp paired-end read lengths. Then, the raw data were used to assemble the complete cp genome using GetOrganelle software (Jin et al. 2020). Gene annotation was performed by the pipeline PGA (Qu et al. 2019) with *S. oligotrichus* (MK994794) as a reference, then coupled with manual adjustment using Geneious v.10.1.3 (Kearse et al. 2012). Analysis of boundaries between IRs and single copy regions was performed by online program GeSeq (Tillich et al. 2017). Finally, the annotated complete cp genome of *S. erythrotrichus* was submitted to NCBI GenBank (accession number: MZ434958).

The complete plastid genome of *S. erythrotrichus* was a circular molecule of 160,731 bp in length. It had a typical quadripartite structure including one large single-copy (LSC) region (85,483 bp), one small single-copy (SSC) region (17,007 bp), and two copies of inverted repeat regions (IRs) (26,780 bp). The overall GC content was 36.90%, while the corresponding values of the LSC, SSC, and IR regions were 34.70%, 30.40%, and 42.50%, respectively. A total of 128 genes were encoded, of which 113 were unique and 17 were duplicated in the IR regions. Among the unique genes, 79 were protein-coding genes, 30 were tRNA genes, and four were rRNA genes.

To explore the phylogenetic position of *S. erythrotrichus* across the Melastomataceae, complete plastid genomes of *S. erythrotrichus* and other 12 species of *Phyllagathis* and *S. oligotrichus* (MK994794) were selected to conduct analyses, using *Tigridiopalma magnifica* (NC_036021.1) as outgroups (Figure 1). The sequences were aligned by MAFFT 7.475 (Rozewicki et al. 2019). The ML analyses were performed with IQ-TREE 1.6.12 (Chernomor et al. 2016). Node support was assessed by 1000 fast bootstrap replicates. Our result indicated that both *Phyllagathis* and *Scorpiothyrsus* are monophyletic, and *S. erythrotrichus* is sister to *S. oligotrichus* (Figure 1).

**Figure 1.**
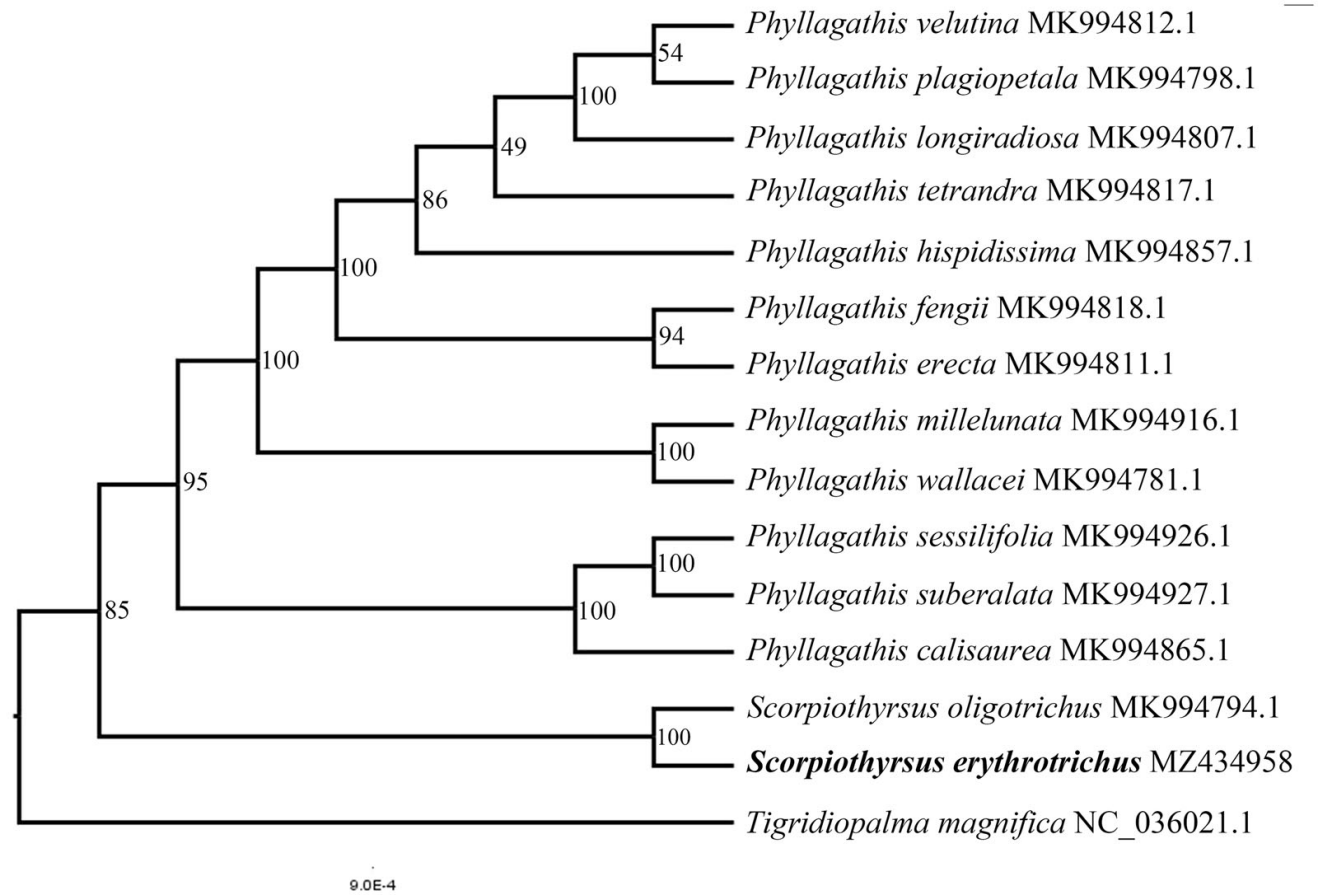
Phylogenetic tree based on 15 complete sequences of chloroplast genome in different species. The position of *Scorpiothyrsus erythrotrichus* is highlighted in bold.

The total genomic DNA was extracted from fresh leaves according to the methods described by Doyle and Doyle (1987) and sequencing was carried out by the Illumina pair-end technology. Raw reads were filtered using NGS QC Toolkit. Clean reads were first aligned to *Calanthe triplicata* (GenBank accession no. NC_024544) and *Calanthe davidii* (GenBank accession no. NC_037438). Filtered reads were then assembled into contigs in the software Platanus version 1.2.4. The physical map of the new chloroplast genome was generated using OGDRAW. Finally, the validated complete cp genome sequence was submitted to GenBank with accession number MN577470.

The fresh leaves of *Annamocarya sinensis* were collected from Xichou county of Yunnan province. Total genome DNA was extracted with the Ezup plant genomic DNA prep kit (Sangon Biotech, Shanghai, China). The voucher specimens of *A. sinensis* were deposited at the herbarium of Southwest Forestry University (accession number: SWFU-YAB-H-0160), and DNA samples were properly stored at Key Laboratory of State Forestry Administration on Biodiversity Conservation in Southwest China, Southwest Forestry University, Kunming, China. Total DNA was used to generate libraries with an average insert size of 350 bp, which were sequenced using the Illumina HiSeq X platform. Approximately, 14.0 GB of raw data were generated with 150 bp paired-end read lengths. Then, the raw data were used to assemble the complete cp genome using GetOrganelle software with *Juglans nigra* as the reference. Genome annotation was performed with the program Geneious R8 (Biomatters Ltd., Auckland, New Zealand) by comparing the sequences with the cp genome of *Juglans nigra*. The tRNA genes were further confirmed through online tRNAscan-SE web servers. A gene map of the annotated *A. sinensis* cp genome was drawn by OGdraw online.

DNA with good integrity and purity was used for library construction and sequencing with the Illumina Hiseq-2500 (San Diego, CA).

## Data Availability

The genome sequence data that support the findings of this study are openly available in GenBank of NCBI at https://www.ncbi.nlm.nih.gov/ under the accession MZ434958. Raw Illumina data were deposited in the NCBI Sequence Read Archive (SRA: SRR14879285, BioProject: PRJNA739828, and Bio-Sample: SAMN19812452).

## References

[CIT0001] Chen C, Renner SS. 2007. Melastomataceae. In: Wu ZY, Raven PH, Hong DY, editors. Flora of China. Vol. 13. Beijing/St. Louis: Science Press/Missouri Botanical Garden Press; p. 360–399.

[CIT0002] Chernomor O, von Haeseler A, Minh BQ. 2016. Terrace aware data structure for phylogenomic inference from supermatrices. Syst Biol. 65(6):997–1008. 27121966 10.1093/sysbio/syw037 PMC5066062

[CIT0013] Doyle JJ, Doyle JD. 1987. A rapid DNA isolation procedure for small quantities of fresh leaf tissue. Phytochem Bull. 19:11–15.

[CIT0003] Hansen C. 1992. The genus *Phyllagathis* (Melastomataceae): characteristics; delimitation; the species in Indo-China and China. Bull Mus Natl D’Hist Nat B Adansonia Bot Phytochim. 14:355–428.

[CIT0004] Jin JJ, Yu WB, Yang JB, Song Y, dePamphilis CW, Yi TS, Li DZ. 2020. GetOrganelle: a fast and versatile toolkit for accurate de novo assembly of organelle genomes. Genome Biol. 21(1):241. 32912315 10.1186/s13059-020-02154-5 PMC7488116

[CIT0005] Kearse M, Moir R, Wilson A, Stones-Havas S, Cheung M, Sturrock S, Buxton S, Cooper A, Markowitz S, Duran C, et al. 2012. Geneious Basic: an integrated and extendable desktop software platform for the organization and analysis of sequence data. Bioinformatics. 28(12):1647–1649. 22543367 10.1093/bioinformatics/bts199 PMC3371832

[CIT0006] Li HL. 1944. Studies in the Melastomataceae of China. J Arnold Arboretum. 25:1–42.

[CIT0007] Merrill ED, Chun WY. 1940. Additions to our knowledge of the Hainan flora. Sunyatsenia. 5:45–200.

[CIT0009] Qu XJ, Moore MJ, Li DZ, Yi TS. 2019. PGA: a software package for rapid, accurate, and flexible batch annotation of plastomes. Plant Methods. 15(1):50. 31139240 10.1186/s13007-019-0435-7 PMC6528300

[CIT0010] Rozewicki J, Li S, Amada KM, Standley DM, Katoh K. 2019. MAFFT-DASH: integrated protein sequence and structural alignment. Nucleic Acids Res. 47(W1):W5–W10. 31062021 10.1093/nar/gkz342 PMC6602451

[CIT0011] Tillich M, Lehwark P, Pellizzer T, Ulbricht-Jones ES, Fischer A, Bock R, Greiner S. 2017. GeSeq – versatile and accurate annotation of organelle genomes. Nucleic Acids Res. 45(W1):W6–W11. 28486635 10.1093/nar/gkx391 PMC5570176

[CIT0012] Zhou Q‐J, Lin C‐W, Dai J‐H, Zhou R‐C, Liu Y. 2019. Exploring the generic delimitation of *Phyllagathis* and *Bredia* (Melastomataceae): a combined nuclear and chloroplast DNA analysis. J Syst Evol. 57(3):256–267.

